# PD-L1 Expression in TNBC: A Predictive Biomarker of Response to Neoadjuvant Chemotherapy?

**DOI:** 10.1155/2017/1750925

**Published:** 2017-12-14

**Authors:** Bruna Cerbelli, Angelina Pernazza, Andrea Botticelli, Lucio Fortunato, Massimo Monti, Paolo Sciattella, Domenico Campagna, Federica Mazzuca, Maria Mauri, Giuseppe Naso, Paolo Marchetti, Giulia d'Amati, Leopoldo Costarelli

**Affiliations:** ^1^Department of Radiological, Oncological and Pathological Sciences, Sapienza, University of Rome, Rome, Italy; ^2^Oncology Unit, Sant'Andrea Hospital, Sapienza University of Rome, Rome, Italy; ^3^Department of Surgery, San Giovanni-Addolorata, Rome, Italy; ^4^Department of Surgical Sciences, Sapienza University of Rome, Rome, Italy; ^5^Department of Statistical Sciences, Sapienza University of Rome, Rome, Italy; ^6^Department of Pathology, San Giovanni-Addolorata, Rome, Italy; ^7^Department of Oncology, San Giovanni-Addolorata, Rome, Italy; ^8^Oncology Unit, Sapienza University of Rome, Rome, Italy

## Abstract

Triple negative breast cancer (TNBC) has an aggressive clinical behaviour, with a poorer prognosis compared to other subtypes. Recently, tumor-infiltrating lymphocytes (TILs) have been proposed as a predictive biomarker for a better clinical outcome and pathological response (pR) after neoadjuvant chemotherapy (NACT) in TNBC. These data confirm the role of the immune system in the neoplastic progression and in the response to therapy. We performed a retrospective analysis of 54 pre-NACT biopsies of TNBC and compared both the percentage of stromal TILs and the degree of PD-L1 expression with the extent of pR to standard NACT. A pathological complete response (pCR) was achieved in 35% of cases. Univariate analysis showed (i) a significant association between PD-L1 expression in ≥25% of neoplastic cells and the achievement of a pCR (*p* = 0.024); (ii) a significantly higher frequency of pCR in cases showing ≥50% stromal TILs (*p* < 0.001). However in the multivariate analysis only PD-L1 expression on tumor cells remained significantly associated with pCR (OR = 1,13; 95% CI 1,01–1,27), suggesting that the expression of this biomarker could be associated with a subpopulation of TNBC more likely to respond to chemotherapy. These data need to be confirmed by larger studies.

## 1. Introduction

Triple negative breast cancer (TNBC) accounts for 10–20% of all breast cancers [1]. It is often associated with high histological grade, presence of lymphocytic infiltration, high rate of distant metastasis, and a poorer prognosis when compared to other breast cancer subtypes. TNBC is generally treated with standard chemotherapy regimens, including both anthracyclines and taxanes, either in the metastatic, adjuvant, or neoadjuvant setting. Neoadjuvant chemotherapy [NACT] is increasingly used in the management of this BC subtype, with pathologic complete response (pCR) rate ranging from 30% to 50% [2–4]. These data point to the need of biomarkers that could be useful to identify the subset of patients more prone to achieve a pCR. In recent reports the presence of tumor-infiltrating lymphocytes (TILs) has been shown to predict the response of TNBC to NACT [5–7]. Moreover, a high number of stromal TILs is predictive of a more favorable outcome in this BC subset. These data underscore the crucial role of the immune system both in the neoplastic progression and in the response to therapy and support the robustness of biomarkers of tumor-immune system interplay in clinical practice [8]. The interaction between programmed cell death protein 1 (PD-1) and its ligand (PD-L1) represents a mechanism of immune escape and a therapeutic target for poor-prognosis malignancies, such as melanoma and non-small-cell lung cancer (NSCLC) [9]. PD-1 is a transmembrane protein of 40 kDa expressed on CD8+ and CD4+ T cells, natural killer (NK) cells, B cells, activated monocytes, and dendritic cells [10]. It is a negative regulator of the immune system that functions by forming a complex with its ligands (either PDL1 or PDL2). Only limited and contrasting data on the role of PD-L1 in breast cancer have been reported so far. In fact, the expression of this marker has been correlated with either a worst [11] or a better prognosis [12].

Apart from their prognostic or predictive value, the presence of stromal TILs and the expression of PD-L1 are strong markers of immune activation in breast cancer and could be involved in the response to preoperative systemic treatment. In this study we aimed to investigate the role of PD-L1 expression and stromal TILs in predicting the pathological response to NACT in TNBC. We retrospectively analyzed 54 pre-NACT biopsies and compared both the percentage of stromal TILs and the extent of PD-L1 expression on neoplastic and inflammatory cells with the effect of neoadjuvant chemotherapy.

## 2. Materials and Methods

Between January 2011 and December 2016, 54 consecutive patients with TNBC received standard NACT (4 cycles of doxorubicin + cyclophosphamide Q3W followed by 12 cycles of paclitaxel weekly) at our Institutions. Clinical information, including age, clinical stage at diagnosis, type of surgery, and pathologic response, was extracted from the institutional databases.

### 2.1. Evaluation of Stromal Tumor-Infiltrating Lymphocytes

Pre-therapy biopsies were retrieved from the Pathology Departments at the Sapienza University Teaching Hospital and the San Giovanni-Addolorata Hospital. Hematoxylin-eosin stained slides were blindly re-evaluated for the presence of stromal tumor-infiltrating lymphocytes (TILs) according to a previously published method [13]. Briefly, TILs were quantified as a percentage of the stromal area of the tumor and expressed as a continuous parameter.

### 2.2. Evaluation of PD-L1 Expression and Immunophenotyping of the Inflammatory Infiltrate

Serial sections were obtained from each paraffin block for (i) immunohistochemical evaluation of PD-L1 expression on both neoplastic and inflammatory cells and (ii) immunophenotyping of the inflammatory infiltrate. PD-L1 immunostains were performed with one of the antibody clones approved for diagnostic assay (SP142, rabbit IgG, dilution 1 : 200, catalog #M4420; Spring Bioscience, Pleasanton, CA) [14] at 1 : 100 dilution, using an automated immunostainer (Benchmark XT, Ventana Medical System, Tucson, AZ, USA) with the Optiview DAB IHC detection kit (Ventana Medical Systems, Tucson, Arizona, USA) according to manufacturer's instructions. Relevant positive controls (human tonsils and placenta) were used for each run of staining. Negative controls were obtained by omitting the primary antibody. The expression of PD-L1 was evaluated separately on all tumor cells and inflammatory infiltrates. A minimum of 200 neoplastic cells were present in each biopsy sample. A positive stain was defined as the presence of membrane staining, either strong or weak, complete or incomplete, in a percentage of cells ≥ 1%, that is, the threshold reported for clinical response to PD-L1 inhibitors in non-small-cell lung carcinoma and has also been reported in breast carcinoma [15, 16]. For each biopsy, both the intensity of membrane staining (scored as 1+ weak, 2+ moderate, and 3+ strong) and the percentage of positive neoplastic cells were recorded, while only the percentage of positive inflammatory cells was evaluated.

Immunophenotyping of the inflammatory infiltrates was carried out with the following antibodies: CD3 for T lymphocytes (Roche, 1 : 100); CD4 (1 : 40) for the helper T subset; CD8 for the cytotoxic T subset (1 : 100); CD20 for B lymphocytes (1 : 200) CD68 for macrophages (1 : 100), and N-CAM (1 : 100) (all from Novocastra, Newcastle, UK). Four images at 20x original magnification (accounting for one mm^2^ of tumor field) were acquired from the areas of maximum inflammatory infiltrate by the NIS Elements Viewer mounted on a Zeiss Axioskop 2 microscope. The number of positive cells/mm^2^ for each antibody was then manually counted on the acquired images.

### 2.3. Evaluation of the Pathologic Response to NACT

The degree of pathologic response of each patient to NACT was obtained from the pathology reports. A complete response was defined as the complete disappearance of invasive tumor cells from breast tissue and regional lymph nodes, regardless of the presence of residual ductal carcinoma in situ (ypT0/is, ypN0) [17, 18].

### 2.4. Statistical Analysis

In the descriptive analysis, quantitative variables were described as mean and range, while qualitative variables were reported as number and percentage. Univariate associations between clinicopathological features and pCR were evaluated using the *χ*2 test or Fisher's exact test, when appropriate. To take into account the effects of all variables on pCR, multivariate analysis were performed by a multivariate logistic regression to estimate the adjuster Odds Ratios (ORs). Statistical significance was set at *p* < 0.05. All analyses were performed using SAS 9.4 (SAS Institute Inc., Cary, NC, USA).

## 3. Results

Clinicopathological features of the 54 patients are detailed in Table 1. Briefly, the mean age at diagnosis was 50 years (range 28–75). In 87% of cases the pre-NACT tumor diameter was larger than 2 cm. Axillary node involvement, assessed by echography and confirmed by fine needle aspiration cytology, was present in 24 patients (45%). The most common histologic subtype was ductal carcinoma of no special type (94% of cases). All tumors were of high nuclear grade (G3) with a proliferation index ≥ 50% in the large majority of cases (74%).

After NACT 30 patients (55%) underwent mastectomy and 24 (45%) had conservative breast surgery, A pCR was achieved in 19 patients (35%).

### 3.1. Tumor-Infiltrating Lymphocytes and Immunophenotype of the Inflammatory Infiltrates in Pre-NACT Biopsies

**Figure 1:**
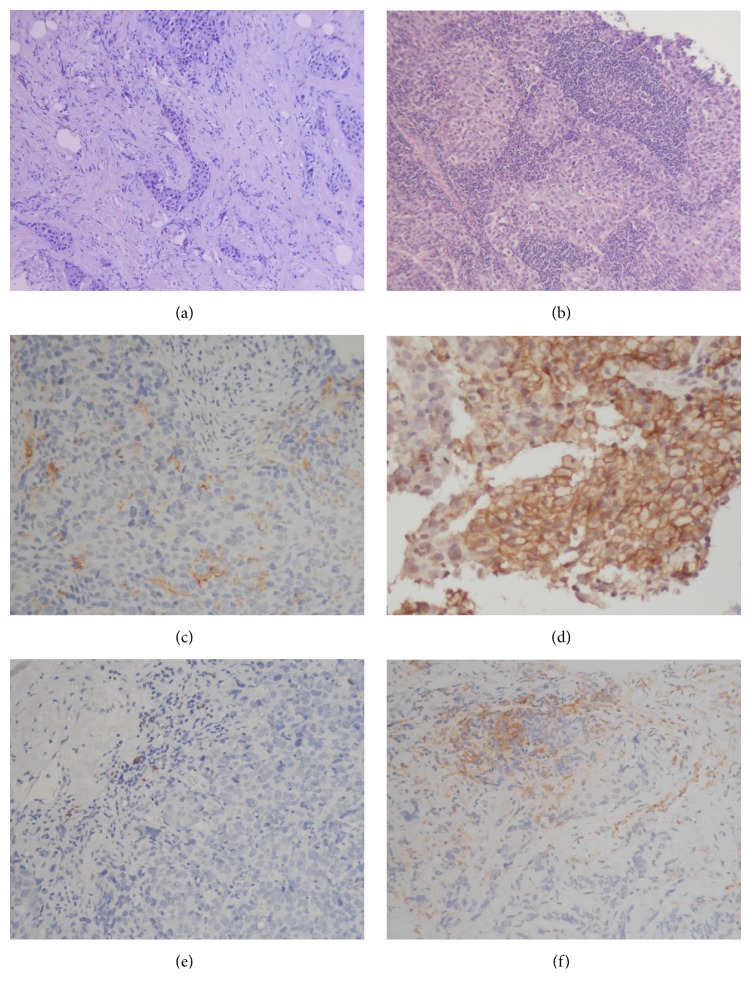
*Evaluation of stromal TILs and PD-L1 expression in TNBC core biopsies*. (a)-(b): low (a) and high (b) level of stromal tumor-infiltrating lymphocytes (haematoxylin and eosin, original magnification ×10). (c)-(d): membranous PD-L1 stain in scattered (c) and diffuse neoplastic cells (d) (PD-L1 immunohistochemical stain, original magnification ×20). (e)-(f): membranous PD-L1 stain in scattered (e) and diffuse inflammatory cells (f) (PD-L1 immunohistochemical stain, original magnification ×20).

The results of stromal TILs evaluations are detailed in Table 2. Briefly, stromal TILs were present in 51 pre-NACT biopsies (95%), with percentages ranging from 2 to 80% (Figures 1(a) and 1(b)). Twenty-four cases (45.5%) had 50% or greater stromal TILs (high TILs) and were accordingly classified as lymphocyte predominant breast cancer (LPBC) [13]. Immunophenotyping of the inflammatory infiltrates revealed a predominance of CD3+ T cells in all biopsy samples. In 36 biopsies (70%) the most represented was the CD8+ subset, followed by the CD4+ subset (27%). Intriguingly, NK cells were absent in 96% of biopsies.

### 3.2. Expression of PD-L1 on Neoplastic and Inflammatory Cells

Membrane staining of neoplastic cells was present in 19 pre-NACT biopsies (35%), with an extent ranging from 1 to 90% (Figures 1(c) and 1(d)). In over 95% of these biopsies (18/19) the percentage of PD-L1 positive tumor cells did not exceed 50%. The membrane stain scored 3+ in 8/19 cases (42%), 2+ in 5/19 (26%), and 1+ in 6/19 (32%). PD-L1 staining of inflammatory cells was present in the majority of biopsies (44 cases, 81%) (Figures 1(e) and 1(f)).

Univariate analysis showed a significant association between the presence of high stromal TILS and the expression of PD-L1 on ≥25% of tumor cells (*p* = 0.008) and ≥10% of inflammatory cells (*p* = 0.002); this association was independent from the prevalent lymphocyte subset (CD8+ or CD4+), and from the CD8/ CD4 ratio.

### 3.3. Comparison of Histologic and Immunohistochemical Data with the Response to NACT

A pCR was achieved in 19 patients (35%). Univariate analysis (Table 2) showed a significant association between the expression of PD-L1 in ≥25% of neoplastic cells and pCR (*p* = 0.02). The presence of pCR was also significantly more frequent in cases showing features of LPBC (with high TILs) in the pre-NACT biopsies (*p* < 0.001). Moreover, pCR was achieved in 100% of patients showing both high TILs and expression levels of PD-L1 ≥ 25% in neoplastic cells in the pre-NACT biopsies (*p* = 0.011, Table 3).

At multivariate analysis (Table 4), only PD-L1 expression on tumor cells remained significantly associated with pCR (*p* = 0.038) with OR of 1,13 (95% CI 1,01–1,27).

## 4. Discussion

The immune system is strongly involved both in the tumor surveillance and in the pathogenesis of breast cancer. Moreover, preexisting immunity against tumor cells is a crucial factor that influences the response to chemotherapy. It is now believed that preexisting antitumor immunity is activated or enhanced during the initial cycle of chemotherapy. During the subsequent cycles, together with incoming acquired drug resistance of the tumor cells, the onset of immune resistance mechanisms impairs the efficacy of treatment [19, 20].

Due to the high histological grading and mutational load, along with the activation of large amounts of genes implicated in immune function, TNBC seems to be the subtype more likely associated with immune system involvement. Thus, the identification of novel immunological prognostic and predictive biomarkers would be useful to guide the choice of the most appropriate treatment, as well as the optimal timing of surgery, especially in the neoadjuvant setting.

Either the presence of stromal TILs or the expression of PD-L1 is being actively investigated as prognostic biomarkers in TNBC. This subset of breast cancer has an aggressive clinical and biological behaviour, with higher risk for early recurrences and a poorer prognosis as compared to the other BC subtypes. However, in the neoadjuvant setting, the achievement of a pCR after NACT is associated with long-term survival. In this study, we investigated the hypothesis that the presence of high stromal TILs and the expression of PD-L1, both markers of immune activation in the tumor microenvironment, could be associated with the rate of pCR in TNBC.

We evaluated pre-NACT core biopsies, which proved to be qualitatively and quantitatively adequate for our analysis. In our study population, 35% of patients achieved a pCR, which is in line with recently published literature [21, 22].

On microscopic evaluation of pre-NACT core biopsies, tumor cell expression of PD-L1 was observed in 35% of cases, although at low levels (≥1% <25% in 15/19 biopsies, 79%). Our observation on a pure sample of TNBC confirms the results of Dill et al. [16] who analyzed a large number of BC with various histologic subtypes, showing the highest rate of PD-L1 expression (32%) in TNBC, with only 5% with diffuse expression on tumor cells (>50%).

PD-L1 expression, both on neoplastic and inflammatory cells, was significantly associated with high stromal TILs. Our observation extends the results reported by Mori et al. [23], which showed a significant association between PD-L1 expression on tumor cells and percentage of stromal TILs on surgical breast specimens, and confirms the parallel behaviour of these immune biomarkers in TNBC. The limited amount of published reports in pure cohorts of TNBC seems to suggest a favorable prognostic role of PD-L1, despite some discrepancies. Mori et al. [23] demonstrated that the interaction between TILs and PD-L1 correlates with a better clinical outcome. However, when high PD-L1 expression is associated with low levels of stromal TILs the prognosis is poor [24]. In the study of Beckers et al. [25] PD-L1, although associated with a better outcome, failed to show an independent prognostic role in this subset of tumors. These partial discrepancies could be explained by differences in the choice of clinical outcomes, in the methods of evaluation of PD-L1 expression on neoplastic cells (membranous versus cytoplasmic) and the cut-off values adopted, and in the antibodies used and the type of sample evaluated (core biopsies versus surgical samples).

There are only limited data on the predictive value of these two biomarkers in TNBC. We found that in this breast cancer subtype the concomitant expression of stromal TILs and PD-L1 on tumor cells membranes was significantly associated with pCR. According to our results, a cut-off of PD-L1 membrane expression on ≥25% of neoplastic cells in pre-neoadjuvant biopsies predicted pCR for TNBC, regardless of staining intensity. On the contrary, the predictive role of TILs showed only a limited power and no statistical association on multivariate analysis. In light of the preliminary results of the KEYNOTE 173 phase II trial [26], reporting a 90% pCR rate in TNBC treated in this setting with the adjunct of pembrolizumab to standard chemotherapy, we hypothesize that TNBC expressing PD-L1 in less than 25% of tumor cells could represent the subset most likely to benefit from this association.

Immunophenotyping of tumor inflammatory microenvironment revealed an excess of CD8+ with a ratio of CD8/CD4 > 1, in line with previous reports [27, 28], although this observation did not reach statistical significance probably due to our limited sample size. Additionally, we found negligible amounts of NK cells in pre-NACT biopsies, although we cannot exclude the fact that their level could have been increased after the first cycle of chemotherapy due to tumor cells death and the release tumor associated antigens.

In conclusion, we showed that a cut-off value of PD-L1 in ≥25% of tumor cells predicts pCR in TNBC and to our knowledge our study is the first dealing with an exclusive population of TNBC cases. A possible explanation for our observation is that PD-L1 expression could be associated with a subpopulation of TNBC with a more aggressive behaviour, likely to respond to chemotherapy. Further studies with larger number of cases are warranted to confirm our findings.

## Figures and Tables

**Table 1:** Clinicopathological features of the study population.

Characteristics	Number of patients (%)
Age (y)	
≤50	30 (55%)
>50	24 (45%)
Pre-NACT tumor size (cT)	
≤2 cm	7 (13%)
>2 cm	47 (87%)
Pre-NACT nodal status (cN)	
Positive	24 (45%)
Negative	30 (55%)
Histotype	
Ductal	51 (94%)
Lobular	1 (2%)
Others	2 (4%)
Nuclear grade	
Grades 1-2	0
Grade 3	54 (100%)
Ki-67	
<50%	14 (26%)
≥50%	40 (74%)
Post-NACT surgery	
Mastectomy	30 (55%)
Segmental mastectomy	24 (45%)
Complete pathological response (pCR) to NACT	19 (35%)

**Table 2:** Association between stromal TILs, the expression of PD-L1 on tumor cells and inflammatory infiltrate, Ki-67 value, cT, cN, and pCR in the univariate analysis.

	*N* (%)	pCR	
		*N*°	*p* value
Stromal TILs			
Absent/low	32 (59%)	*8*	*<0.001*
High	22 (41%)	*11*	

PD-L1 on tumor cells			
0%	35 (65%)	*11*	
≥1–<25%	15 (28%)	*4*	
≥25%	4 (7%)	*4*	*0.024*

PD-L1 on inflammatory cells			
Negative	10 (19%)	*3*	*ns*
Positive	44 (81%)	*15*	

Ki-67			
<50%	14 (26%)	*4*	*ns*
≥50%	40 (74%)	*15*	

cT			
T1	7 (13%)	*3*	*ns*
T2–T4	47 (87%)	*16*	

cN			
Negative	30 (56%)	*13*	*ns*
Positive	24 (44%)	*6*	

**Table 3:** The achievement of pCR according to levels of both stromal TILs and PD-L1 expression on neoplastic cell membranes (low TILs/low PD-L1; high TILs/low PD-L1; low TILs/high PD-L1; high TILs/high PD-L1).

		PD-L1 on neoplastic cells				
TILs		<25%		≥25%		
	pt	pt	*pCR*	pt	*pCR*	
Low	32	32	25%	0		*p* = 0.011
High	22	18	39%	4	100%	

**Table 4:** Association between the expression of PD-L1 on neoplastic cells and inflammatory infiltrate, stromal TILs, Ki67, clinical T, clinical N, and pCR in the multivariate analysis.

	*N* (%)	pCR		
		*N* (%)	*p* value	ORR (CI)
Stromal TILs				
Low	32 (60%)	8 (25%)	0.5	1,61 (0,40–6,52)
High	22 (40%)	11 (50%)		

PD-L1 on tumor cells				
0%	35 (65%)	11 (31%)	0.038	1,13 (1,01–1,27)
1–25%	15 (28%)	4 (27%)		
≥25%	4 (7%)	4 (100%)		

PD-L1 on inflammatory cells				
Negative	10 (18%)	3 (30%)	0.058	0,09 (0,01–1,08)
Positive	44 (82%)	15 (34%)		

Ki-67				
<50%	14 (26%)	4 (28%)	0.054	1,05 (1–1,09)
≥50%	40 (74%)	15 (37%)		

Clinical T				
T1	7 (13%)	3 (43%)	0.8	0,8 (0,08–8,09)
T2–T4	47 (87%)	16 (34%)		

Clinical N				
Negative	30 (55%)	13 (43%)	0.27	0,47 (0,12–1,82)
Positive	24 (45%)	6 (25%)		
